# Improving Adherence to AAP Acute Otitis Media Guidelines in an Academic Pediatrics Practice through a Quality Improvement Project

**DOI:** 10.1097/pq9.0000000000000553

**Published:** 2022-06-14

**Authors:** Ryan M. Wolf, Kyle T. Langford, Barron L. Patterson

**Affiliations:** From the * Department of Pediatrics, Monroe Carell Jr. Children’s Hospital at Vanderbilt, Nashville, Tenn.; † Vanderbilt University School of Medicine, Nashville, Tenn.

## Abstract

**Introduction:**

Acute otitis media (AOM) is the most common reason for antibiotic use in children. The American Academy of Pediatrics (AAP) published its latest AOM guidelines in 2013. A safety-net antibiotic prescription (SNAP) is recommended for some patients based on age, severity, and duration of symptoms. At baseline, 78% of patients diagnosed with AOM in our general pediatrics practice met AAP guidelines, and 20% of eligible patients received a SNAP according to guidelines. We aimed to increase adherence to AAP AOM guidelines in an academic general pediatrics clinic from 78% to 90% by January 2020.

**Methods:**

A quality improvement team determined key drivers and developed interventions. Patients included were 6 months to 12 years old with AOM. Encounters were reviewed for adherence to AAP AOM guidelines. During the project, interventions included an ear pain note template, which generated guideline-based recommendations, note template education in clinic orientation sessions, a didactic session on AOM management, and reminders on workstations. Data were analyzed using P-charts.

**Results:**

Percent of AOM encounters (n = 1266) adhering to AAP AOM guidelines increased from 78% to 92%. We also reviewed two process measures. First, the use of the ear pain note template increased from 0% to 44%. Second, the percent of AOM encounters where an eligible patient received a SNAP increased from 21% to 78% (encounters n = 421).

**Conclusion:**

We demonstrate increased adherence to AAP AOM guidelines, including improved use of SNAPs after introducing a note template with clinical decision support and provider educational sessions.

## INTRODUCTION

### Background

American children frequently receive antibiotic therapy they do not need. As a result, children are prescribed more than 10,000,000 unnecessary antibiotics in the United States every year.^1^ The outpatient setting is of particular interest to antibiotic stewardship efforts because approximately 60% of antibiotics in the United States are prescribed in ambulatory settings.^2^ Theories behind overprescribing antibiotics include pressure from patients and their families, actual or perceived parental satisfaction, provider knowledge of appropriate management, and time constraints felt by providers.^1,3^

Acute otitis media (AOM) is a common disease in pediatric patients and accounts for 22 million provider visits every year in the United States. The highest incidence of AOM occurs between 6 and 24 months. AOM is commonly treated with antibiotics. The American Academy of Pediatrics (AAP) released recommendations for AOM management in 2004, followed by an update in 2013. These guidelines recommend that watchful waiting with a safety-net antibiotic prescription (SNAP) can be used in certain patients diagnosed with AOM.^4–6^ The release of the AAP AOM guidelines align with greater national efforts to combat unnecessary use of antibiotics in the United States.^6,7^ In one study, 62% of patients given a SNAP never needed to fill the antibiotic prescription.^8^

### Review of Current Evidence

Past work demonstrated overall adherence to AAP guidelines ranging from 40% to 90%, with 34% to 44% of encounters eligible for a SNAP.^9–12^ A SNAP is offered anywhere from 1% to 10% of eligible encounters at baseline.^9–11^ Several prior studies aimed to increase adherence to AAP guidelines through targeted provider education, audit/feedback, and quality improvement (QI) efforts. Sun et al.^11^ showed overall adherence rates of 60% in an emergency department after a wide range of Plan-Do-Study-Act (PDSA) cycles (creation of watchful waiting handouts, educational posters displayed in ED rooms). In a large factorial-design cluster-randomized trial with 24 primary care practices, Forrest et al.^10^ demonstrated that clinical decision support (CDS) coupled with provider performance feedback increased adherence to AOM guidelines but limited provider adoption of SNAPs as a treatment option. Their CDS tool had a broad range of use in practice, ranging from 5% to 45% of visits for AOM. While progress has been made,^12^ we postulate that in our academic practice, a CDS tool in the electronic medical record (EMR) with high use could lead to a sustained increase in adherence to guidelines and a resultant increase in the appropriate use of SNAPs.

### Local Problem

**Fig. 1.**
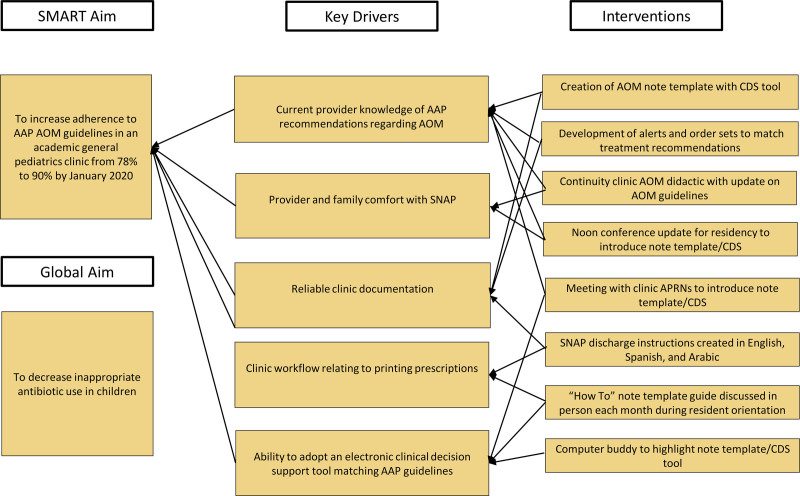
Key driver diagram. SMART, specific, measurable, achievable, relevant, timely.

The setting for our QI project was the Pediatric Primary Care Clinic (PPCC) at Monroe Carell Jr. Children’s Hospital at Vanderbilt, an academic general pediatrics practice in Nashville, Tennessee. Baseline data in our practice showed adherence with AAP AOM treatment guidelines 78% of the time, and SNAP use only 21% when recommended by AAP guidelines. Therefore, the team developed a SMART (specific, measurable, actionable, relevant, and timebound) aim statement to increase providers’ adherence to established AAP guidelines for the management of AOM from 78% to 90% and to increase the use of SNAP when indicated from 21% to 75% by January 2020. A QI team consisting of a provider leader, resident leader, medical student, PPCC leadership, clinic advanced practice registered nurses, and resident stakeholders met regularly to review the SMART aim and progress following the Model for Improvement framework.^13^ We identified key drivers (Fig. 1) and used serial PDSA cycles while implementing interventions.

## METHODS

### Ethical Issues

The institutional review board reviewed the protocol and determined that, as QI work, it did not qualify as human research.

### Setting

PPCC serves as the medical home for 18,900 pediatric patients, and care is provided by resident physicians, attending physicians, and advance practice registered nurses. In addition, the clinic serves as the continuity clinic for 72 general pediatrics residents. It provides acute, ambulatory experiences for an additional 24 medicine-pediatrics residents and dozens of medical students each year. There are approximately 20,000 acute visits and an additional 22,000 health maintenance visits in a typical year. Every academic year, resident physicians spend 4 weeks assigned to the clinic caring for acute visit concerns. Two supervisory attending physicians staff acute visits and oversee six residents and two advance practice registered nurses each day. TennCare, Tennessee’s version of Medicaid, insures approximately 80% of patients in the clinic. More than 40% of encounters are performed in languages other than English. PPCC uses Epic as its EMR (Epic Systems, Verona, Wis.).

### Developing Interventions

Before this project, there were no formal antibiotic stewardship programs in the ambulatory clinics at our institution. However, clinic providers were accustomed to alerts within the EMR to aid in the rapid completion of common tasks such as treating anemia or streptococcal pharyngitis. No CDS existed that guided actual treatment decision-making. We identified AOM as a disease process amenable to a CDS due to its common presentation and guideline-based care in collaboration with clinic leadership. Recognizing our low usage of SNAPs led us to test, develop, and implement CDS based on the 2013 AAP guidelines on managing healthy children with uncomplicated AOM.

### Note Template and CDS

**Fig. 2.**
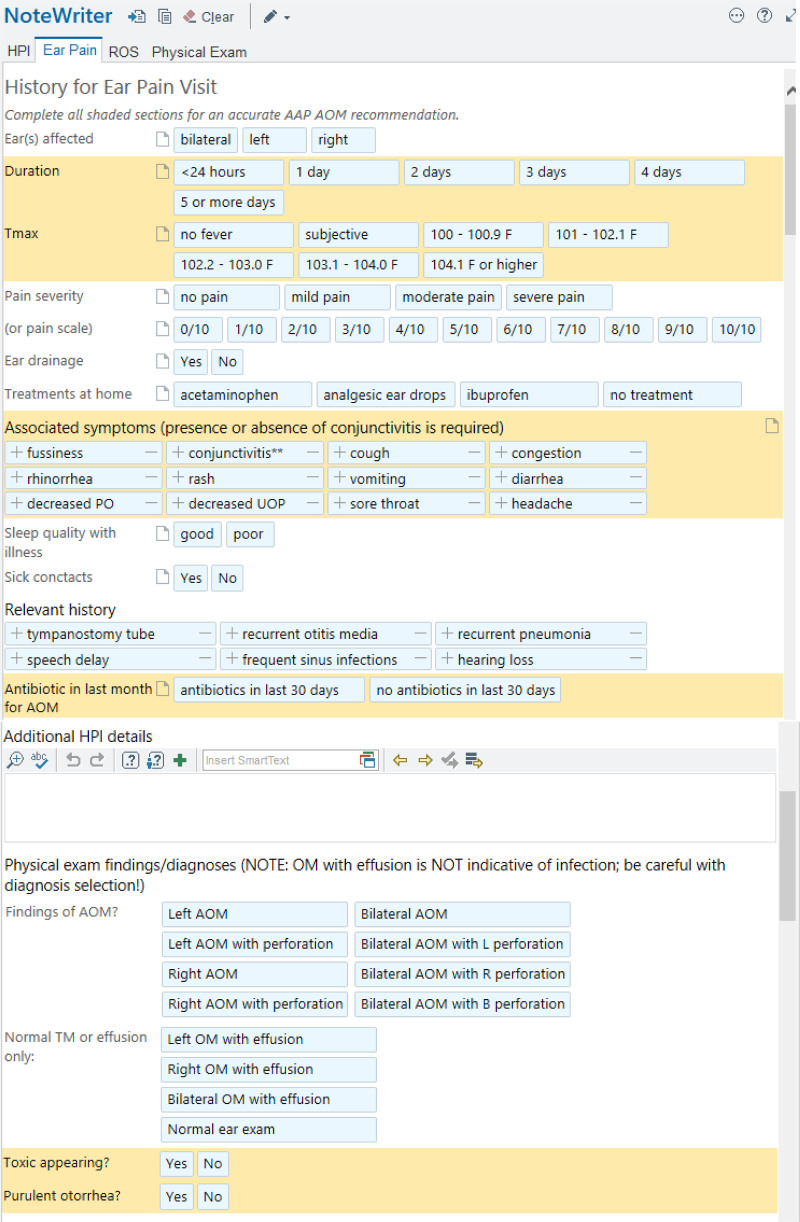
CDS tool/note template. Printed with permission from © 2021 Epic Systems Corporation.

Before introducing this project, providers used a standard “acute clinic visit” note template that included sections for history, review of systems, physical examination, and an assessment/plan. Much of the note was free text. We created an “ear pain” note template (Fig. 2) that collected the discrete data to calculate treatment recommendations based on AAP guidelines. This note template could easily be incorporated into an ongoing or well-child check note when ear pain was not the chief complaint. These guidelines primarily assess patient age, severity/duration of otalgia, maximum temperature, otorrhea, and the presence of unilateral or bilateral AOM to aid in treatment choice.^5^ The history of present illness section of the visit note captures these historical data points. A general review of systems area included a mandated box to check for the presence of conjunctivitis. Additionally, we included a checkbox to indicate the use of antibiotics in the last 30 days. The physical examination section included expandable pictures of mild, moderate, and severe AOM for learners to view and referenced treatment tables from the AAP guidelines.^6^ Selecting items in the note template also generated text for the clinic note to speed clinical documentation.

After entering all required elements in the ear pain note template, the EMR generated one of five treatment recommendations: amoxicillin, amoxicillin/clavulanic acid, cefdinir, a SNAP, or symptomatic treatment for otalgia only. Based on the CDS recommendation of the ear pain template, an EMR alert presented the provider with an order set based on the associated recommendation. The order sets included any recommended antibiotic prescription (automatically sent to the patient’s pharmacy electronically if treatment with immediate treatment with antibiotics was recommended or printed on paper if a SNAP was recommended), options for antipyretics/analgesics, specific discharge instructions based on treatment recommendations and patient language, and an appropriate ICD-10 diagnosis code. For example, if a patient had a documented penicillin allergy in the EMR, the CDS would recommend cefdinir when appropriate. If a patient had been treated with amoxicillin for AOM in the past 30 days, the CDS would recommend amoxicillin/clavulanic acid when appropriate.

### Interventions

(1) The QI team introduced the project to all members of the acute clinic team at PPCC. Additional interventions included: (2) unveiling of the note template to advance practice registered nurses during a multi-disciplinary meeting, (3) leading a didactic session with all residents on AOM management (4) providing ongoing note template/CDS education during monthly clinic orientation sessions for new residents before rotating on the service, (5) updating to CDS based on provider feedback, (6) adding discharge instructions translated to Spanish, and (7) placing reminders to use note template on clinic workstations.

### Measures

Our QI project evaluated the primary effect of implementing CDS tools followed by several PDSA cycles by assessing three outcomes over time. The primary outcome measure was adherence to AAP guidelines on AOM for all encounters with a diagnosis of AOM (29 unique ICD-10 diagnosis codes of H66 including suppurative, unilateral, bilateral, bullous myringitis, perforation, etc.). Specifically, we evaluated adherence to recommendation AAP AOM 3A/3B regarding appropriate indications for antibiotic therapy in children diagnosed with AOM, recommendations 3C/3D regarding observation without use of antibacterial agents, and recommendations 4A/4B regarding amoxicillin treatment as the primary treatment of choice when treatment is indicated^5^ (Table 1). Process measures included the percentage of encounters eligible for a SNAP where a SNAP was given, and the percentage of AOM encounters using the CDS tools. As a balancing measure, all patients given a SNAP were assessed for provider messages and clinic or emergency room visits related to AOM within 7 days of their initial encounter.

**Table 1.** Key AAP 2013 Recommendations for AOM^5^ Used for Outcome Measures

3A	The clinician should prescribe antibiotic therapy for AOM (bilateral or unilateral) in children 6 mo and older with severe signs or symptoms (ie, moderate or severe otalgia or otalgia for at least 48 h, or temperature 39 °C [102.2°F] or higher)
3B	The clinician should prescribe antibiotic therapy for bilateral AOM in children younger than 24 mo without severe signs or symptoms (ie, mild otalgia for less than 48 h, temperature less than 39 °C [102.2°F])
3C	The clinician should either prescribe antibiotic therapy *or* offer observation with close follow-up based on joint decision-making with the parent(s)/caregiver for unilateral AOM in children 6 to 23 mo of age without severe signs or symptoms (ie, mild otalgia for less than 48 hours, temperature less than 39 °C [102.2°F]). When observation is used, a mechanism must be in place to ensure follow-up and begin antibiotic therapy if the child worsens or fails to improve within 48 to 72 h of onset of symptoms
3D	The clinician should either prescribe antibiotic therapy *or* offer observation with close follow-up based on joint decision-making with the parent(s)/caregiver for AOM (bilateral or unilateral) in children 24 mo or older without severe signs or symptoms (ie, mild otalgia for less than 48 h, temperature less than 39 °C [102.2°F]). When observation is used, a mechanism must be in place to ensure follow-up and begin antibiotic therapy if the child worsens or fails to improve within 48 to 72 h of onset of symptoms
4A	Clinicians should prescribe amoxicillin for AOM when a decision to treat with antibiotics has been made *and* the child has not received amoxicillin in the past 30 d *or* the child does not have concurrent purulent conjunctivitis *or* the child is not allergic to penicillin
4B	Clinicians should prescribe an antibiotic with additional β-lactamase coverage for AOM when a decision to treat with antibiotics has been made *and* the child has received amoxicillin in the past 30 d *or* has concurrent purulent conjunctivitis *or* has a history of recurrent AOM unresponsive to amoxicillin

### Measurement and Analysis

A retrospective chart review was used to obtain all data. Baseline data were obtained from December 19, 2018, to April 16, 2019. A gap in data collection from April 17, 2019, to June 5, 2019, occurred due to a delay in implementing the CDS tool. The CDS tool intervention became widely available on June 5, 2019, with data obtained through June 5, 2020. During the intervention period, we reviewed 1,863 encounters with a diagnosis of AOM ages 6 months to 12 years. Review of clinical documentation from the EMR for age, presence of otorrhea, unilateral or bilateral AOM, duration/severity of otalgia, temperature, and antibiotic or SNAP usage assessed for adherence to guidelines and eligibility for SNAP (Table 1). Adherence was a bundled measure, meaning all criteria were needed to count as adherent to AAP recommendations. We excluded patients not seen in PPCC for their original diagnosis of AOM (n = 68) or if their chart did not contain sufficient information to identify adherence to AAP recommendations (n = 52) accurately. Two study authors (R.W. and K.T.L.) reviewed the data to assess adherence to guidelines using a standardized chart review checklist for each encounter. Encounters with questionable notation were reviewed together and decided upon mutually. Data were plotted every two weeks on a P-chart created using QI Charts add-in for Microsoft Excel. We used established special cause rules to detect nonrandom signals of change within the data. For example, after eight consecutive points were noted above previously established centerlines, an upward shift in our mean centerlines occurred.^13^

## RESULTS

### Baseline Data

Retrospective baseline data review took place from December 2018 to April 2019 (n = 477). Of these 477 patients, 126 were eligible for management with a SNAP (26%). Of these eligible patients, 26 (21%) were given a SNAP. At baseline, 78% of encounters with patients diagnosed with AOM adhered to AAP management guidelines.

### Intervention Data

**Fig. 3.**
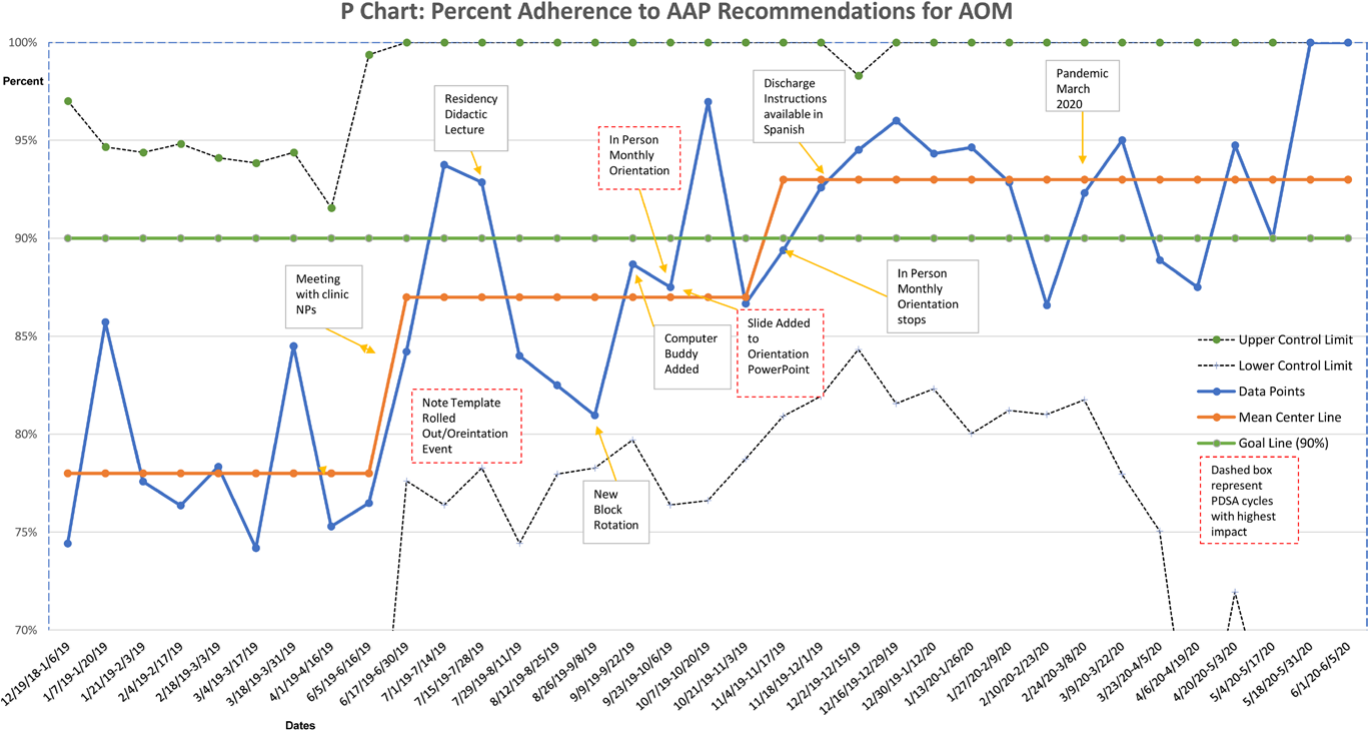
P control chart showing the percent of all encounters for AOM with adherence to the AAP recommendations and various PDSA cycles at points in time.

**Fig. 4.**
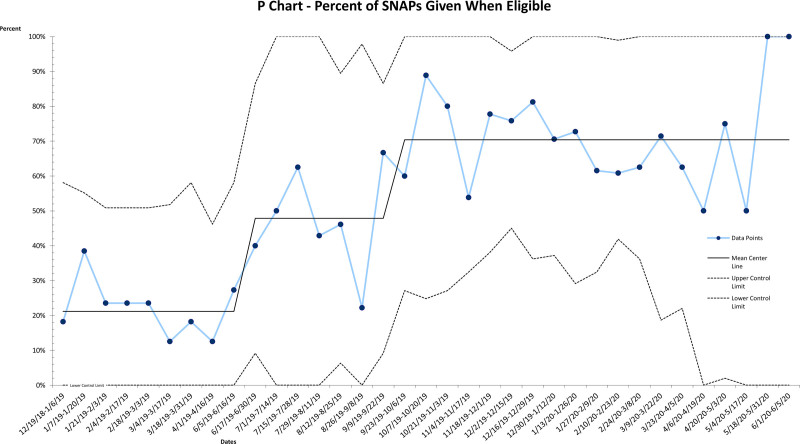
P control chart showing the percentage of encounters when a SNAP is eligible and prescribed.

**Fig. 5.**
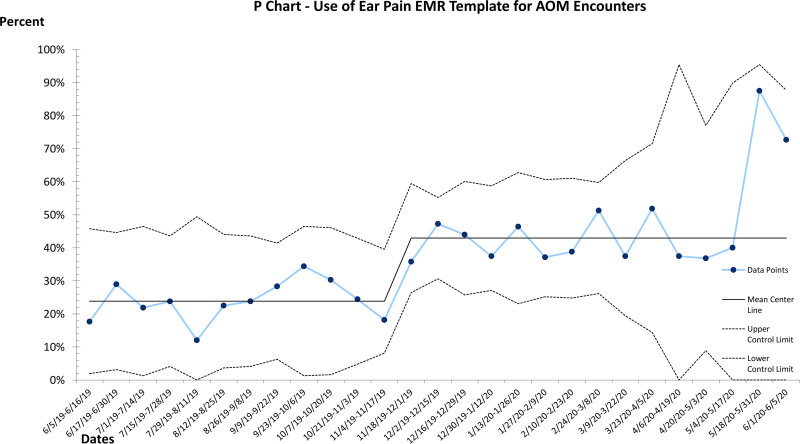
P control chart showing the percentage of encounters when the AOM note template is used.

During the intervention phase from June 2019 to June 2020, there was an average of 23 visits with a diagnosis of AOM per week (total n = 1,266). After PDSA cycles 1–3 from June 2019 to September 2019, adherence to AAP guidelines for all visits for AOM shifted from 78% to 85%. These PDSA cycles focused on provider education of the note template and education of the management of AOM. However, we noticed a downward trend of all three measures after each new block rotation which correlated to trainee rotation change and a new group of trainees joining PPCC. PDSA cycles 4–7 cycles focused on a monthly orientation for trainees and incorporation of provider feedback on the note template and patient education materials. From September 2019 to December 2019, adherence shifted from 85% to 92% (Fig. 3). Similar shifts occurred along the same timeline for the percentage of SNAP given during eligible encounters: from a baseline of 21% to 45% to 74% (Fig. 4). From June 2019 to June 2020, providers prescribed 187 SNAPs. Two shifts also occurred with provider note template usage. Again, we started at a baseline of 0%. From June 2019 to October 2019, note template usage was at 22% of all encounters. It then shifted to 44% of all encounters (total n = 455) (Fig. 5).

In March 2020, the COVID-19 pandemic resulted in dramatically lower acute clinic visits. There was an average of nine episodes of AOM weekly from March 2020 to June 2020, compared to 29 episodes weekly from June 2019 to February 2020. Due to lower volumes, we chose to plot our data every 2 weeks rather than weekly. This change also resulted in wider upper and lower control limits during that time. This QI project also demonstrated the effect of this particular CDS tool on AOM management. Providers prescribed a SNAP 96% of the time when the CDS tool recommended a SNAP. After achieving our goal adherence of ≥90%, we measured data for another six months from January 2020 to June 2020 to demonstrate sustained improvement.

### Balancing Measure

From June 2019 to June 2020, providers prescribed 187 SNAPs. Of those, 4.8% (n = 9) were seen in clinic, the emergency department, or sent a message to their provider related to their AOM within seven days of the initial encounter: three patients sent a message for continued fever; three patients sent a message for continued otalgia; one patient went to the emergency department for continued fever, and two patients returned to the clinic for continued fever. All were instructed during those encounters to start their SNAP.

## DISCUSSION

### Interpretation

This study demonstrated embedding CDS into the workflow for AOM increases adherence to AAP guidelines for AOM and the use of SNAP. We accomplished this through several various interventions. A key innovation was to build an easily integrated CDS to make a recommendation based on AAP guidelines in real-time within existing provider workflows. This intervention builds upon prior work by Forrest et al. and their CDS tool in the primary care setting and the QI work performed by Sun et al. in the ED. These authors’ educational interventions led to behavioral change and increased adherence to guidelines. Our primary outcome measure of AAP AOM adherence exceeded that of Sun et al. (92% compared to 60%). By using QI techniques, education, and CDS, we believe our interventions were successful due to ease of use, transparency of decision-making, incorporation into clinic workflow, and appropriate patient-facing materials, particularly related to the use of SNAPs. Another advantage may be our primary care academic setting, where providers are accustomed to participating in QI projects. Our baseline adherence to AAP guidelines was higher than the previously described baselines. It may be that newly trained resident physicians can more easily incorporate existing guidelines for AOM management. In a clinic with short-term, high turnover resident physician staffing, our process of CDS was uniquely suited to lead to long-lasting change. Audit/feedback cycles are helpful interventions, but the frequency of providers rotating through PPCC monthly prompted a more system-wide change.

Although not all providers at PPCC used the note template for AOM encounters, it seems our general educational PDSA interventions contributed to increasing adherence to AAP guidelines whether or not the note was used. Future analysis should identify barriers to using the CDS during encounters and why providers rarely disregard CDS recommendations.

### Limitations

There were several limitations with this project. First, our setting was in an academic practice where overall adherence to AAP guidelines was relatively high at baseline, which may not generalize to other practices. Chart review was retrospective, and we had to rely on clinical documentation as it existed to make determinations about adherence to guidelines. A gap in chart review occurred before the implementation of the project. To our knowledge, there was no institutional intervention focused on AOM antibiotic stewardship during the gap. Third, we tracked adherence data as a group rather than the individual provider. We did not differentiate note template usage between resident providers, attending providers, or advanced practice registered nurses. We were not able to determine if pharmacies filled SNAPs or not. Finally, although this study suggests the long-term sustainability of SNAP, our study’s timespan was limited to one academic year. We did not track data across academic years with the arrival of new trainees and the departure of former senior residents.

## CONCLUSIONS

Using a note template with embedded CDS and order sets for AOM based on AAP guidelines coupled with ongoing provider education effectively increased adherence to guidelines in this academic pediatric practice. Six months following the completion of PDSA cycles, adherence to guidelines and use of SNAPs continued to be maintained. We believe similar CDS could be used in other pediatric clinical settings (urgent care clinics, emergency rooms, and other general pediatric practices) and for other clinical conditions. These efforts may help avoid unnecessary antibiotic prescribing behaviors in the future.

## DISCLOSURE

The authors have no financial interest to declare in relation to the content of this article.
